# *TNF-α* G-308A genetic variants, serum CRP-hs concentration and DNA damage in obese women

**DOI:** 10.1007/s11033-019-04764-0

**Published:** 2019-03-21

**Authors:** Marta Włodarczyk, Michał Ciebiera, Grażyna Nowicka

**Affiliations:** 1 Department of Biochemistry and Pharmacogenomics, Faculty of Pharmacy with Division of Laboratory Medicine, Medical University of Warsaw, Banacha 1B, 02-097 Warsaw, Poland; 2 Centre for Preclinical Research, Medical University of Warsaw, Banacha 1B, 02-097 Warsaw, Poland; 3 II Department of Obstetrics and Gynecology, The Centre of Postgraduate Medical Education, Cegłowska 80, 01-809 Warsaw, Poland

**Keywords:** DNA damage, Obesity, CRP, TNF gene, Polymorphism

## Abstract

Obesity is associated with inflammation, which can disturb genome stability. Tumor necrosis factor (*TNF-α*) polymorphism was found to affect TNF-α protein production and inflammation. Therefore, the present study illustrates the relationship between *TNF-α* polymorphism, the degree of inflammation assessed by serum high sensitivity C-reactive protein concentration (CRP-hs) and basal DNA damage in patients with obesity (BMI 30–34.9 kg/m^2^) and control subjects with proper body mass (BMI < 25 kg/m^2^). A total of 115 participants (75 obese premenopausal women; and 40 age-, and gender-matched controls) were included. Biochemical parameters (serum concentrations of total-cholesterol, HDL-cholesterol, LDL- cholesterol, triglycerides, glucose, apolipoprotein AI, CRP-hs) and endogenous DNA damage (determined by comet assay) were measured. *TNF-α* G-308A polymorphism (rs1800629) was analyzed by PCR-RFLP (PCR-restriction fragments length polymorphism). An effect of *TNF-α* genotype on serum CRP-hs concentration was noted (*p* = 0.031). In general, carriers of the rare A allele of the *TNF-α* G-308A polymorphism had significantly lower endogenous DNA damage and serum CRP-hs concentrations than GG homozygotes, however, the protective effect of the A allele was especially visible in non-obese women. Serum CRP-hs concentrations and levels of DNA damage (% DNA in tail) were significantly higher in obese than in controls (*p* = 0.001 and *p* < 0.0001, respectively*)*. The adjusted multiple linear regression analyses revealed a significant, independent impact of obesity on DNA damage (*p* = 0.00000) and no effect of other covariates i.e. age, *TNF-α* genotype and serum CRP-hs concentration. Our study showed that obesity has a significant impact on the levels of endogenous DNA damage. Obesity abolished the protective effect of A allele of the *TNF-α* G-308A polymorphism on DNA damage and on inflammation development observed in non-obese A allele carriers.

## Introduction

Smoking, improper diet and environmental toxins have been recognized as main exogenous sources of DNA damage [1]. However, besides exogenous factor-caused DNA breaks, endogenous DNA damage and failure of DNA repair can disturb cell metabolism and function [2]. In cells, production of reactive oxygen species (ROS) and inflammation have been recognized to cause DNA lesions [3]. DNA damage has been found to be involved in aging and development of common diseases including cancer, atherosclerosis, metabolic syndrome [4–6].

The Comet Assay is a sensitive and low-cost technique, which measures DNA damage in individual cells [7, 8]. In addition to DNA strand breaks (double strand breaks and single strand breaks), the modified Comet Assay serve to detect also oxidized bases, interstrand cross-links or misincorporated uracil [9]. This method is widely used to determine the level of DNA damage, both resulting from exposure to environmental mutagens, as well as arising in the course of many diseases [10–12].

Obesity is a worldwide problem with increasing prevalence, associated with co-morbidities such as type 2 diabetes mellitus and cardiovascular diseases, and increased cancer risk [13–15]. Obesity is characterized by the, adipocyte hypertrophy, elevated production of reactive oxygen species, cytokines, chronic inflammation, disturbances in insulin and glucose metabolism [16–18]. Association between the occurrence of DNA lesions and enhanced body weight has been also reported [19–21]. Both inflammation and metabolic disturbances can cause DNA damage [22–24]. Moreover, the relationship between chronic inflammation and genomic instability has been observed in about 25% of human cancers [25–27].

Tumor necrosis factor-alpha (TNF-α) is a multi-functional cytokine synthesized by adipocytes, preadipocytes, endothelial cells, smooth muscle cells, fibroblasts, leukocytes and macrophages [28–31]. It can participate in regulation of many cellular processes such as immune function, differentiation, proliferation, apoptosis and energy pathways [32, 33]. Variations in the *TNF-α* gene can affect TNF-α production and a significant effect of the polymorphism in the promoter region of the *TNF-α* gene at position -308 (rs1800629) was reported [34–36]. Presence of the variant allele has been shown to increase the rate of transcription and production of the TNF-α protein [37–39]. G-308A polymorphism in the *TNF-α* gene has been associated with the development of inflammation and risk of cardiovascular diseases [40–44]. A recent study revealed the predisposition of GG homozygotes to higher production of pro-inflammatory molecules resulting in their enhanced serum levels [45]. The G-308A polymorphism was also found to be associated with insulin sensitivity and increased production of leptin, suggesting an impact of *TNF-α* gene on obesity and obesity-related health complications [46]. Phillips et al. showed that patients carrying the GG genotype had elevated risk of metabolic syndrome compared with carriers of the minor A allele [47]. However, large cohort studies in Chinese, Caucasians and Afro-Americans did not show a significant correlation between G-308A polymorphism and insulin resistance or obesity [48–51]. Recently published systematic review and meta-analysis have indicated an association between *TNF-α* G-308A gene polymorphism and the risk of ischemic heart disease [28, 52].

TNF-α stimulates the production of C-reactive protein (CRP) and the development of inflammatory processes, and serum CRP concentration is commonly used as a marker of inflammation [53–56]. Furthermore, in vitro studies showed increased DNA damage as a result of TNF-α stimulated ROS production [57]. TNF-α together with IL-1β and IFN-γ induced DNA damage in human cholangiocarcinoma cell line [58]. DNA damage and enhanced ROS levels were related to TNF-α—mediated senescence in HUVEC (human umbilical vein endothelial cells) [59].

The aim of our study was to assess the impact of the G-308A *TNF-α* polymorphism on CRP-hs levels and genomic stability measured by basal DNA damage in obese women.

## Materials and methods

### Subjects

The study participants were premenopausal women (Polish Caucasians from the Warsaw region). Among 115 participants of the study, 75 were obese. Obesity was classified according to World Health Organization criteria [60] i.e., subjects with BMI ≥ 30 kg/m^2^ were considered obese. The obese women were consecutively recruited between December 2011 and June 2013 on the basis of clinical assessments from subjects who had been directed to the Outpatient Clinic at the National Food and Nutrition Institute in Warsaw due to obesity treatment. The gender- and age-matched control group (n = 40) of apparently healthy women with proper body mass (BMI not exceeded 25 kg/m^2^) was recruited from subjects directed for a routine general health screening. The study was conducted according to the guidelines laid down in the Declaration of Helsinki and the Local Ethics Committee at the National Food and Nutrition Institute approved all procedures involving human subjects. Written informed consent was obtained from all of the registered volunteers.

The recruited women were premenopausal, non- smoking (for at least 5 years), had no history of alcoholism, and had no signs or symptoms of renal and hepatic disorders, endocrine disorders (e.g. disease of the thyroid, parathyroid, Cushing’s syndrome, polycystic ovary syndrome), autoimmune diseases, and cancer. Women within the last 3 months before the study were not receiving medications known to influence plasma lipid levels and did not use hormonal therapy as well as did not report chronic use of dietary supplements and anti-inflammatory drugs. Exclusion factors were also menopause, pregnancy, and lactation.

### Anthropometric measurements

All subjects underwent a comprehensive medical evaluation including medical history, physical examination and measurement of anthropometric parameters: body weight, body height, waist circumference, hip circumference according to standardized procedures routinely performed in the Outpatient Clinic at the National Food and Nutrition Institute (Warsaw, Poland). The body waist circumference was measured at the midpoint between the lower margin of the last rib cage and the top iliac crest by using a flexible inch tape. Measurements were taken in the morning, after an overnight fasting, at the same day, or the day before blood samplings. Based on anthropometric measurements the BMI and WHR (waist-hip ratio) indexes were calculated.

### Blood analysis

Blood was collected after night fasting from all subjects and serum parameters were analyzed on the same day. Total cholesterol, HDL-cholesterol, triglycerides, glucose, and insulin were measured using standard techniques in a certified laboratory for clinical chemistry at The National Food and Nutrition Institute. The LDL cholesterol levels were calculated using the Friedewald formula. Residue serum was aliquoted and frozen at − 20 °C until analysis. CRP-hs concentrations were obtained using commercially available ELISA (Immundiagnostik AG, Germany), according to the protocol provided by the manufacturer. The serum concentrations of apolipoprotein AI were measured using monoclonal antibodies against apolipoprotein AI (Pointe Scientific, USA) by the immunotubidymetric method.

### Genotype analysis

Genomic DNA was extracted from peripheral white blood cells of whole-blood samples using DNA Mini Kit (A&A Biotechnology, Poland). Extracted DNA samples (100 ng) were amplified to obtain a fragment including the polymorphic region of *TNF-α* G-308A gene (rs 1,800,629), as previously described [61] by using the following primers: F5′-AATAGGTTTTGAGGGCCATG-3′ and R5′-GGGACACACAAGCATCAAGG-3′. Approximately 100 ng of DNA was amplified by thermal cycling using the DNA polymerase kit (BioLine, London, UK) in 25 *µ*L of PCR mixture containing 2.5 mM MgCl_2_, 0.4 mM of each deoxyribonucleotide triphosphate (dNTP, New England Biolabs, USA), 1 U *Taq*, and 100 pM of each primer. Polymerase chain reaction conditions included an initial denaturation at 94 °C for 5 min followed by 35 cycles of 94 °C for 15 s, 55 °C for 30 min, and 72 °C for 15 s, with a final extension at 72 °C for 7 min. The amplified DNA samples containing a polymorphic site was digested with the restriction enzyme *NcoI* (New England Biolabs, USA) and products were run on agarose gel electrophoresis. Digestion of the 151 bp fragment carrying the G allele was giving 139 bp and 12 bp fragments, while the fragment with the A allele remained intact. About 20% of all samples were randomly selected for repeated genotyping for confirmation. Concordance between repeats was 100%.

### Comet assay

DNA integrity was determined by the use of alkaline single-cell gel electrophoresis (comet assay), based on previous reports [62, 63]. Lymphocytes were obtained from 1 mL heparinized blood by centrifugation in a density gradient; then 50 µL of lymphocytes (1–3 × 10^5^ cells/mL) was distributed with 50 µL of 2% low-melting-point agarose on a microscope slide precoated with 0.5% normal agarose. The slides were incubated for 1 h in a freshly prepared cold (4 °C) lysis solution (2.5 M NaCl, 100 mM EDTA-Na_2_, 10 mM Tris, pH 10.0–10.5) with 1% Triton X-100. Next, the slides were left in a horizontal gel electrophoresis tank with alkaline electrophoresis buffer (300 mM NaOH, 1 mM EDTA-Na_2_, pH > 13.0) for 40 min at 4 °C. Electrophoresis was performed under following conditions: 20 min, 35 V (1 V/cm), 300 mA. Slides were then washed with a neutralizing solution (0.4 M Tris, pH 7.5), and stained with DAPI (20 µg/ml).Nikon Eclipse 50i fluorescence microscope (×400 magnification) and Lucia Comet Assay software version 4.81 (Laboratory Imaging, Prague, Czech Republic) was used to analyze 100 comets on each slide. From each subject three blood samples were analyzed in duplicates. Of the data obtained, % DNA in the tail was chosen for further analysis as a DNA damage parameter. The chemicals were supplied by Sigma–Aldrich.

### Statistical analysis

All statistical calculations were performed with the Statistica software (version 12.0). The distribution of variables was tested by Shapiro–Wilk test. Differences in continuous parameters were tested using Mann–Whitney U-test. Spearman correlation analyses were performed for the relationships among the variables. Non-continuous variables were tested with a Chi square test. Allele frequencies for *TNF-α* variants were calculated with the gene counting method. Hardy–Weinberg equilibrium (HWE) was determined by Pearson’s χ^2^ goodness-of-fit test. CRP-hs was dichotomized as ≥ 3 mg/L versus otherwise (< 3 mg/L) based on the well accepted cut-off point (of 3 mg/L) indicating elevated CRP-hs associated with an increased risk for CVD [64]. Regression analyses were performed using generalized linear models. Odds ratios (OR) with 95% confidence intervals (95% CI) were calculated using logistic regression. Results were expressed as means ± SD or percentages, and *p* < 0.05 was considered statistically significant.

## Results

Characteristics of the studied subjects are summarized in Table 1. Obese and control subjects (non-obese) were in similar age. There were substantial differences in serum concentrations of total cholesterol, triglycerides, LDL-cholesterol, and blood pressure between studied groups (*p* < 0.05). While no differences in HDL-cholesterol, apolipoprotein AI and glucose concentrations were found. Serum C-reactive protein (CRP-hs) concentrations as well as mean level of DNA damage (% DNA in tail) were significantly higher in obese than in controls (Table 1).

**Table 1:** Clinical and biochemical characteristics of the study population

	Non-obese (n = 40) mean ± SD	Obese (n = 75) mean ± SD	*p * value
Age (years)	36 ± 10	38 ± 6	0.325
BMI (kg/m^2^)	21.04 ± 1.75	32.73 ± 1.93	0.000
WHR	0.80 ± 0.06	0.88 ± 0.05	0.000
Systolic blood pressure (mmHg)	115.20 ± 11	126.99 ± 18.21	0.000
Diastolic blood pressure (mmHg)	74.17 ± 8.95	82.33 ± 8.46	0.000
Total Cholesterol (mg/dL)	174.20 ± 32.16	202.67 ± 35.07	0.000
HDL-Cholesterol (mg/dL)	61.37 ± 12.42	59.72 ± 15.47	0.549
LDL-Cholesterol (mg/dL)	96.32 ± 28.86	122.20 ± 30.05	0.000
Triglycerides (mg/dL)	88.55 ± 32.89	105.33 ± 43.61	0.030
Glucose (mg/dL)	83.56 ± 8.72	84.77 ± 8.26	0.719
Apolipoprotein AI (mg/dL)	157.02 ± 32.51	158.72 ± 27.61	0.922
CRP-hs (mg/L)	2.96 ± 1.71	4.13 ± 2.26	0.001
DNA damage (%)	1.60 ± 0.44	4.25 ± 1.22	0.000

Data are presented as means ± standard deviations (SD)

*p* value from Mann–Whitney U-test

*BMI* body mass index, *WHR* waist-hip ratio, *CRP-hs* high sensitivity C-reactive protein

Among all studied subjects the distribution of *TNF-α* gene alleles was in Hardy–Weinberg equilibrium (χ^2^ value = 2.68, *p* = 0.100). The frequency of the rare A allele of the *TNF-α* gene G-308A polymorphism was 17.4%. 71% of the studied women carried the GG genotype (wildtype), 24%—the GA genotype and 5%—the AA genotype. As reported in Table 2, no statistically significant difference in frequency of the three genotypes of G-308A *TNF-α* polymorphism among obese and non-obese was found. However, the prevalence of obesity was higher among subjects with GG genotype than among A allele carriers (71% and 50% respectively, *p* = 0.026). Due to the low frequency of the AA genotype statistical analyses were performed for A allele carriers (GA and AA genotypes pooled) and GG homozygotes.

**Table 2:** The distribution of genotypes and alleles of *TNF-α* G-308A polymorphism in obese and non-obese subjects

	Non-obese (n = 40) N (%)	Obese (n = 75) N (%)	*p* value
Genotypes			
GG	23 (57.5%)	58 (77%)	0.0851
GA	14 (35%)	14 (19%)	
AA	3 (7.5%)	3 (4%)	
HWE, *p*	0.674	0.096	
GG	23 (57.5%)	58 (77%)	0.0264
A (AA and GA)	17 (42.5%)	17 (23%)	
Alleles			
G allele	60 (75%)	130 (87%)	0.0262
A allele	20 (25%)	20 (13%)	

*HWE* Hardy–Weinberg equilibrium; *p* value from χ^2^ test

Study participants’ characteristics according to the G-308A *TNF-α* polymorphism are presented in Table 3. In GG homozygotes higher diastolic blood pressure was observed. No statistically significant difference in mean values of BMI and WHR between analyzed groups was recognized. Carriers of the GG genotype had higher levels of DNA damage as well as higher CRP-hs serum concentrations compared to the A allele carriers (3.55 ± 1.70% vs. 2.80 ± 1.29%; *p* = 0.025 and 4.06 ± 2.07 mg/L vs. 2.92 ± 2.15 mg/L, respectively, *p* = 0.0001).

**Table 3:** Clinical and biochemical characteristics of the study population according to *TNF-α* G-308A gene polymorphism

	A allele carriers (n = 34) Mean ± SD	GG genotype carriers (n = 81) Mean ± SD
Age (years)	36 ± 10	37 ± 7
BMI (kg/m^2^)	27.11 ± 6.15	29.3 ± 5.70
WHR	0.83 ± 0.08	0.86 ± 0.05
Systolic blood pressure (mmHg)	120.62 ± 14.80	123.68 ± 17.77
Diastolic blood pressure (mmHg)	77.19 ± 9.50	80.33 ± 9.33*
Total cholesterol (mg/dL)	193.91 ± 38.92	192.28 ± 35.78
HDL-cholesterol (mg/dL)	59.97 ± 11.20	60.43 ± 15.68
LDL-cholesterol (mg/dL)	114.16 ± 32.47	112.79 ± 32.01
Triglycerides (mg/dL)	101.88 ± 41.59	98.49 ± 40.77
Glucose (mg/dL)	85.60 ± 9.59	84.09 ± 7.85
Apolipoprotein AI (mg/dL)	154.40 ± 22.70	159.45 ± 31.36
CRP-hs (mg/L)	2.92 ± 2.15	4.06 ± 2.07**
Tail DNA (%)	2.80 ± 1.29	3.55 ± 1.71***

Data are presented as means ± standard deviations (SD)

*BMI* body mass index, *WHR* waist-hip ratio, *CRP-hs* high sensitivity C-reactive protein

Mann–Whitney U-test: *p = 0.037, **p = 0.0001, ***p = *0.025*

In studied group DNA damage was significantly correlated with BMI, WHR, systolic and diastolic blood pressure as well as total cholesterol, LDL cholesterol, and serum CRP-hs concentrations (Table 4). The observed association between DNA damage and BMI was not affected by the *TNF-α* genotype. However, an impact of the *TNF-α* genotype on the associations between DNA damage and WHR and serum CRP-hs was observed. Only among A allele carriers a strong, positive correlation between DNA damage and WHR was observed (R = 0.784, *p* = 0.00004). Also in A allele carriers but not in GG homozygotes, a positive correlation between DNA damage and serum CRP-hs concentration was recognized (Table 4, R = 0.578, *p* = 0.0003).

**Table 4:** Spearman correlations between DNA damage (% DNA in tail) and biochemical and anthropometric parameters

Variables	All subjects (n = 115) R *p* value	A allele carriers (n = 34) R *p* value	GG genotype carriers (n = 81) R *p* value
Age (years)	0.183 0.0502	0.092 0.605	0.205 0.066
BMI (kg/m^2^)	0.725 0.00000	0.788 0.00000	0.687 0.00000
WHR	0.304 0.0064	0.760 0.00004	0.075 0.579
Systolic blood pressure (mmHg)	0.454 0.00000	0.508 0.003	0.387 0.0004
Diastolic blood pressure (mmHg)	0.381 0.00003	0.288 0.110	0.369 0.0007
Total cholesterol (mg/dL)	0.283 0.0022	0.306 0.078	0.300 0.0067
HDL-cholesterol (mg/dL)	− 0.007 0.941	− 0.314 0.069	0.077 0.491
LDL-cholesterol (mg/dL)	0.273 0.0032	0.304 0.080	0.281 0.011
Triglycerides (mg/dL)	0.162 0.084	0.207 0.239	0.180 0.108
Glucose (mg/dL)	0.162 0.084	0.413 0.070	0.413 0.304
Apolipoprotein AI (mg/dL)	− 0.048 0.706	− 0.150 0.566	− 0.056 0.707
CRP-hs (mg/L)	0.286 0.002	0.578 0.0003	0.177 0.113

*BMI* body mass index, *WHR* waist-hip ratio, *CRP-hs* high sensitivity C-reactive protein

**Fig. 1:**
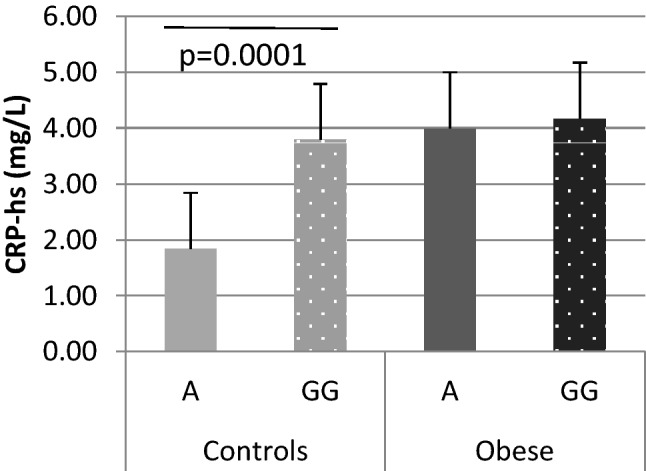
CRP-hs levels among studied groups. A significant difference in CRP-hs levels between GG homozygotes and A allele carriers of *TNF-α* G-308A polymorphism was seen among controls (non-obese) but not among obese

When obese and non-obese carriers of studied genotypes were analyzed separately, obesity not *TNF-α* genotype was found to affect DNA damage as higher levels of DNA damage occurred in obese compared to non-obese. Obese women carrying GG genotype had only slightly more DNA damage (% DNA in tail) than obese A allele carriers (4.37 ± 1.27% and 3.84 ± 0.97%, respectively; *p* = 0.084). Also among non-obese women non-significant allele effect on DNA damage was observed (1.49 ± 0.43% in GG and 1.75 ± 0.41% in A allele carriers; *p* = 0.057). The G-308A *TNF-α* polymorphism was found to affect serum CRP-hs concentrations only in non-obese women. Mean serum CRP-hs concentration was significantly higher in non-obese women with GG genotype than in non-obese A allele carriers (*p* = 0.0001), while among obese participants similar CRP-hs levels were observed (Fig. 1).

Taking into account the degree of inflammation, higher, but not significant incidence of elevated serum CRP-hs concentrations (≥ 3 mg/L) was found among obese subjects: (OR 1.97 95% CI 0.88–4.41, *p* = 0.095). Additionally, logistic regression analysis identified GG genotype as a risk factor for elevated CRP-hs (≥ 3 mg/L) only in non-obese women (Table 5). The odds of CRP-hs ≥ 3 mg/L in non-obese women with GG genotype was 50 times greater than in non-obese women with A allele (*p* = 0.0001, OR 50.00, 95% CI 6.95–359.75). Multiple linear regression analyses revealed also a significant interaction effect of TNF-α genotype and elevated CRP-hs (≥ 3 mg/L) on the levels of DNA damage (F = 4.75, *p* = 0.031) in all studied women. However, when all covariates elevated CRP-hs, TNF-α genotype, obesity and age were included into the statistical analyses, the impact of obesity (BMI ≥ 30 kg/m^2^) on the level of DNA damage was independent of other covariates (F = 69.41, *p* = 0.0000) and no significant interaction effect was observed.

**Table 5:** Relationship between obesity, *TNF-α* polymorphism and elevated CRP-hs concertation in serum (≥ 3 mg/L)

	Crude OR (95% CI); *p*	Age adjusted OR (95% CI); *p*
Obesity (BMI ≥ 30 kg/m^2^)	1.97 (0.88–4.41) p = 0.095	2.04 (0.9–4.61) p = 0.08
*TNF-α* genotype (GG)	6.42 (2.64–15.57) p = 0.00003	6.49 (2.66–15.85) p = 0.00003
Non-obese *TNF-α* genotype (GG)	50 (6.95-359.75) p = 0.0001	56.57 (7.00-456.91) p = 0.0001
Obese *TNF-α* genotype (GG)	2.01 (0.64–6.34) p = 0.227	2.15 (0.66–6.98) p = 0.196

*BMI* body mass index, *CRP-hs* high sensitivity C-reactive protein

## Discussion

Obesity, that is a result of an imbalance between energy intake and expenditure, has reached epidemic proportions with increasing prevalence worldwide. Adipose tissue participates in the production of inflammatory mediators, and in adipose tissue from obese enhanced TNF-α production was observed [51, 52, 65–67].

The G-308A polymorphism in the promoter region of the *TNF-α* gene was found to affect TNF-α protein expression and ischemic heart disease risk in, both, Caucasians and Asians [68, 69]. The human TNF-α protein is coded by the gene located near major histocompatibility complex (MHC) between the class I HLA-B and the class II HLA-DR loci [70, 71]. Therefore, the SNPs in the *TNF-α* promoter may be related to HLA haplotypes and autoimmune diseases such as systemic lupus erythematosus (SLE) and rheumatoid arthritis (RA) [72–74].

Low-grade chronic inflammation is a characteristic feature of obesity, and plays an important role in the pathogenesis of obesity-associated comorbidities [16]. Inflammation is linked with enhanced generation of reactive oxygen species (ROS), which can damage cellular biomolecules, including DNA, leading to disturbances in cell signaling and cell cycle control, genetic mutations, and promotion of inflammation [75].

In vitro studies recognized that pro-inflammatory cytokines provoke DNA damage, cell senescence and growth arrest [59, 76, 77]. In IFNγ/TNFα-induced genotoxicity, NADPH oxidases (Nox 1 and 4) and TGFβ/SMAD pathways are involved in enhanced ROS production [76]. ROS formation and increased level of DNA lesions were observed as a result of high CRP-hs in the culture of HUVECs [78]. Oxidative stress, chronic inflammation and DNA damage have been recognized as important factors leading to the development of carcinogenesis, atherosclerosis and cardiovascular diseases. Obesity is associated with elevated risk of all these diseases [79–83]. Therefore, not only obesity-associated inflammation but also obesity-associated DNA damage may play a significant role in the development of both cardiovascular diseases and cancer in obese [26, 84]. In cancerogenesis, enhanced mutation rate was found to be linked to a high amount of DNA lesions [85, 86].

The G-308A polymorphism in *TNF-α* gene was reported in relation to TNF-α protein production and development of inflammation as well as it was suggested to play an important role in the development and progression of cancer [87–89]. Therefore, this polymorphism may affect both development of inflammation and formation of DNA lesions. Thus, we hypothesized that in obese amount of endogenous DNA lesions may be linked to the degree of inflammation and *TNF-α* gene polymorphism, and in the present study we assessed the association between G-308A *TNF-α* gene variants, serum concentrations of CRP-hs and DNA damage in obese.

The present study was conducted in Caucasian women from the central region of Poland and among our study participants we found the low frequency of the AA genotype (5%) as well as A allele (17.4%) of the G-308A polymorphism in *TNF-α* gene. This is consistent with the results of other studies reporting that the G to A change in *TNF-α* gene is rather rare [90]. In a study of 120 Caucasian Italian women no AA homozygotes were recognized and the frequency of A allele was 27.4% [90]. The frequency of AA genotype was about 4% among Han Chinese [91], 2% among people with obesity from Spain [92], and 0.6% in the Brazilian individuals [93]. In GG homozygotes higher plasma levels of TNF-α and CRP-hs than in AA homozygotes [94–97] as well as in GA heterozygotes of the G-308A polymorphism in the *TNF-α* gene were reported [90].

In the present study non-obese carriers of the A allele had significantly lower CRP-hs serum concentrations than GG homozygotes. The presence of the A allele appears to have a protective anti-inflammatory effect, which, however, disappears when obesity appears. We observed similar CRP-hs concentrations in both obese and non-obese GG homozygotes, while obese had higher levels of DNA damage. It indicates that in obese GG homozygotes other factors than inflammation, have a significant impact on cellular DNA damage. In A allele carriers DNA damage was positively correlated with serum CRP-hs concentration and in obese-A allele carriers similar levels of serum CRP-hs and DNA damage as in obese-GG homozygotes were observed. Our study participants, both obese and controls, can be classified as apparently healthy, thus we can hypothesize that obesity and low-grade inflammation characteristic for obesity can affect basal DNA damage observed in this study. We found significantly greater amount of DNA lesions in obese than in non-obese women as well as the associations between BMI and DNA damage. It is in agreement with our previous study [21]. However, the results of the presented study show that the impact of obesity and obesity-associated disturbances on DNA damage is strong and occurrence of obesity eliminates or significantly decreases the effect of the G-308A *TNF-α* variants on both inflammation, and levels of DNA damage. In obesity oxidative stress and inflammation are involved in the induction of DNA lesions and have an impact on the efficiency of the DNA repair mechanisms [24]. DNA damage in cells may be induced by pro-inflammatory cytokines, chemokines and molecules such as NO (nitric oxide), and ROS [98, 99]. In white adipose tissue amount of DNA damage was related to pro-inflammatory markers such as IL-6 and TNF-α [77]. As CRP-hs exerts ROS production in vitro [78] it can be hypothesized that the potential cause of DNA damage found in our study participants is oxidative stress related to enhanced inflammation (CRP-hs ≥ 3 mg/dl). Our results indicates that in non-obese, apparently healthy women GG homozygosity of the G-308A *TNF-α* polymorphism is associated with enhanced low grade inflammation assessed by serum CRP-hs concentrations, and occurrence of obesity does not affect significantly CRP-hs levels in GG homozygotes. The presence of A allele in non-obese women protects against inflammation but development of obesity abolished this allele effect.

A broad range of DNA lesions has been recognized in people with obesity [100–102]. Enhanced DNA damage was also reported in patients with obesity-related diseases such as type 2 diabetes and metabolic syndrome [103]. Moreover, body weight loss resulted in a reduction in the level of DNA damage [104–107]. The amount of DNA with oxidative damage was associated with levels of cholesterol, triglycerides and HbA1c [108]. In the present study a relationship between DNA damage and both, total cholesterol and LDL-cholesterol was also observed. Our study does have some limitations and one important limitation is a small sample size, which may be responsible for the observed lower frequency of A-allele carriers among obese than among non-obese. Moreover, we studied only women, therefore, data for men as well younger and older populations containing subjects of both genders and BMI in a wide range are needed.

In summary, the present study demonstrates the strong impact of obesity on basal DNA damage assessed by the comet assay (as % DNA in tails) and indicates that the presence of obesity abolished the protective effect of A allele on inflammation development observed in non-obese women.
